# Acute Emergent Biliary Pathologies Identified Using Point-of-Care Ultrasound

**DOI:** 10.3390/diagnostics16183023

**Published:** 2026-09-17

**Authors:** Sahana Ummadi, Tallia Pearson Kroeger, Anil Prasad, Cooper Stinson, Andrew G. Theophanous, Nina Angeles, Denise Elizondo, Rebecca G. Theophanous

**Affiliations:** 1 Department of Emergency Medicine, School of Medicine, Duke University, 2301 Erwin Rd, Durham, NC 27703, USA; sahana.ummadi@duke.edu (S.U.); tallia.pearson@duke.edu (T.P.K.); nina.angeles@duke.edu (N.A.); denise.elizondo@duke.edu (D.E.); 2 School of Medicine, Duke University, 2301 Erwin Rd, Durham, NC 27703, USA; anil.prasad@duke.edu (A.P.); cooper.stinson@duke.edu (C.S.); 3 College of Medicine, University of Toledo, 3000 Arlington Ave, Toledo, OH 43614, USA; atheophanous2@gmail.com; 4 Durham Veterans Affairs Healthcare System, 508 Fulton St, Durham, NC 27710, USA

**Keywords:** biliary ultrasound, point-of-care ultrasound, cholelithiasis, acute cholecystitis, emphysematous gallbladder, choledocholithiasis, gallbladder polyp, biliary neoplasia

## Abstract

Abdominal pain is a common presentation in the emergency department (ED). Ultrasound is the first-line diagnostic imaging modality for right upper quadrant pain. Point-of-care ultrasound (POCUS) is a focused examination that can be performed at the patient’s bedside to evaluate acute and emergent biliary pathology. The advantages of POCUS include avoiding the potential safety risks associated with transporting critically ill patients, no exposure to ionizing radiation, potentially reduced costs, and rapid diagnosis. This narrative review synthesizes key sonographic findings and techniques for emergent and non-emergent biliary diseases that physicians should recognize at first glance. We present significant and interesting images of cholelithiasis, acute cholecystitis, emphysematous gallbladder, choledocholithiasis or sludge, Mirizzi syndrome, post-operative biliary obstruction, gallbladder polyps, biliary neoplasms, and malignant-appearing lesions or masses. Finally, this review integrates POCUS into clinical pathways to guide decisions regarding additional abdominal imaging, such as computed tomography or radiology-performed ultrasound, and surgical consultation.

**Figure 1:**
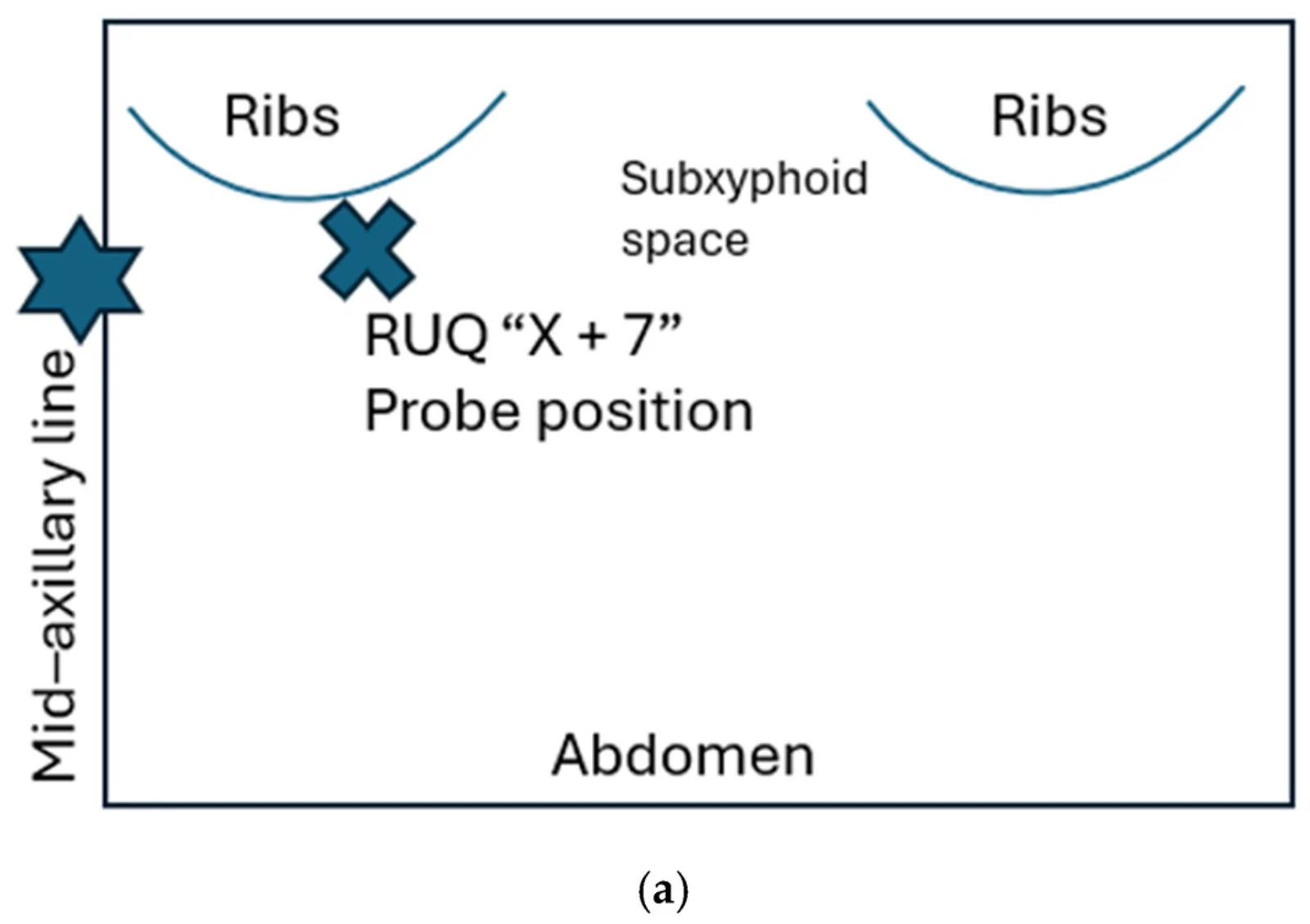

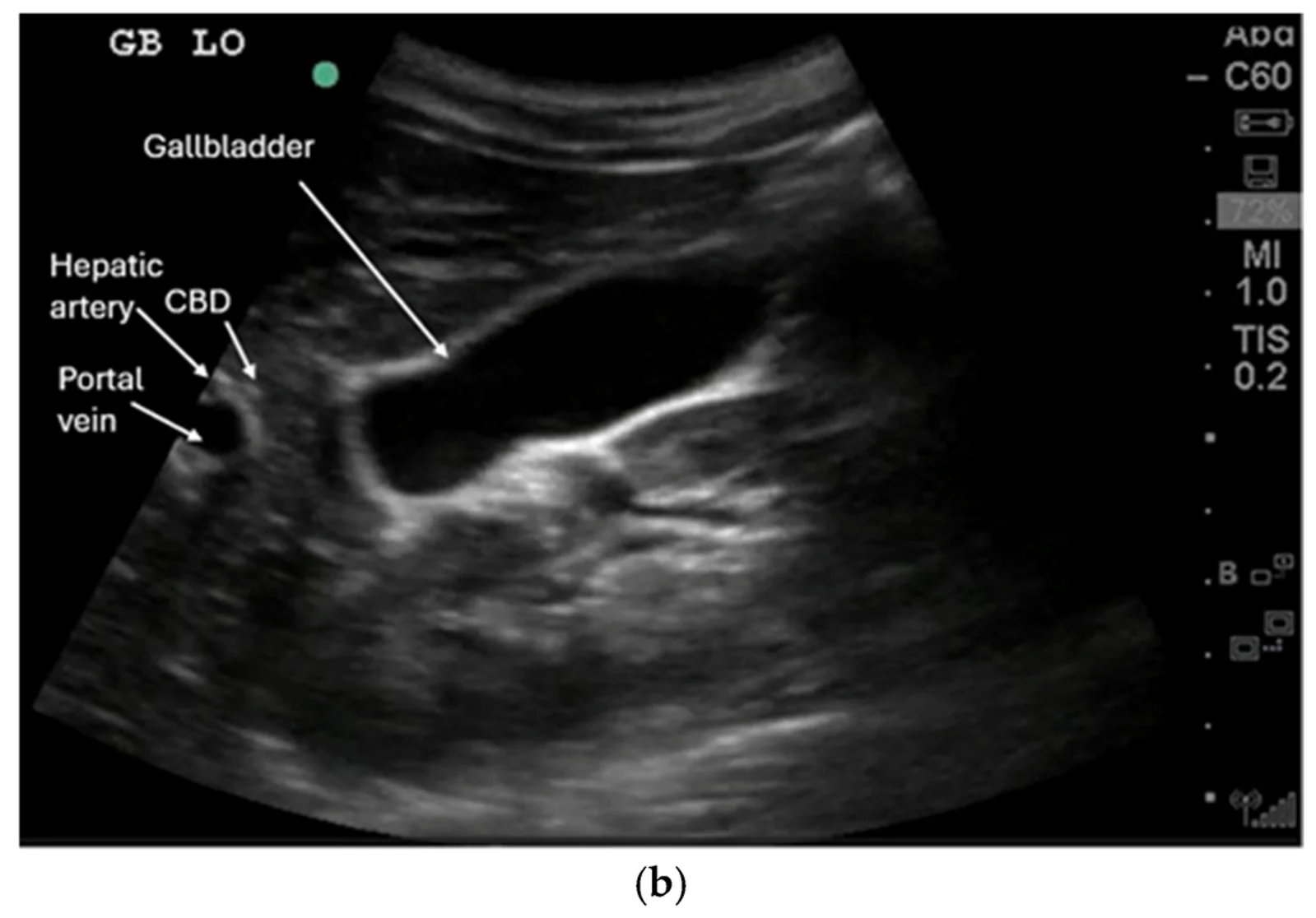

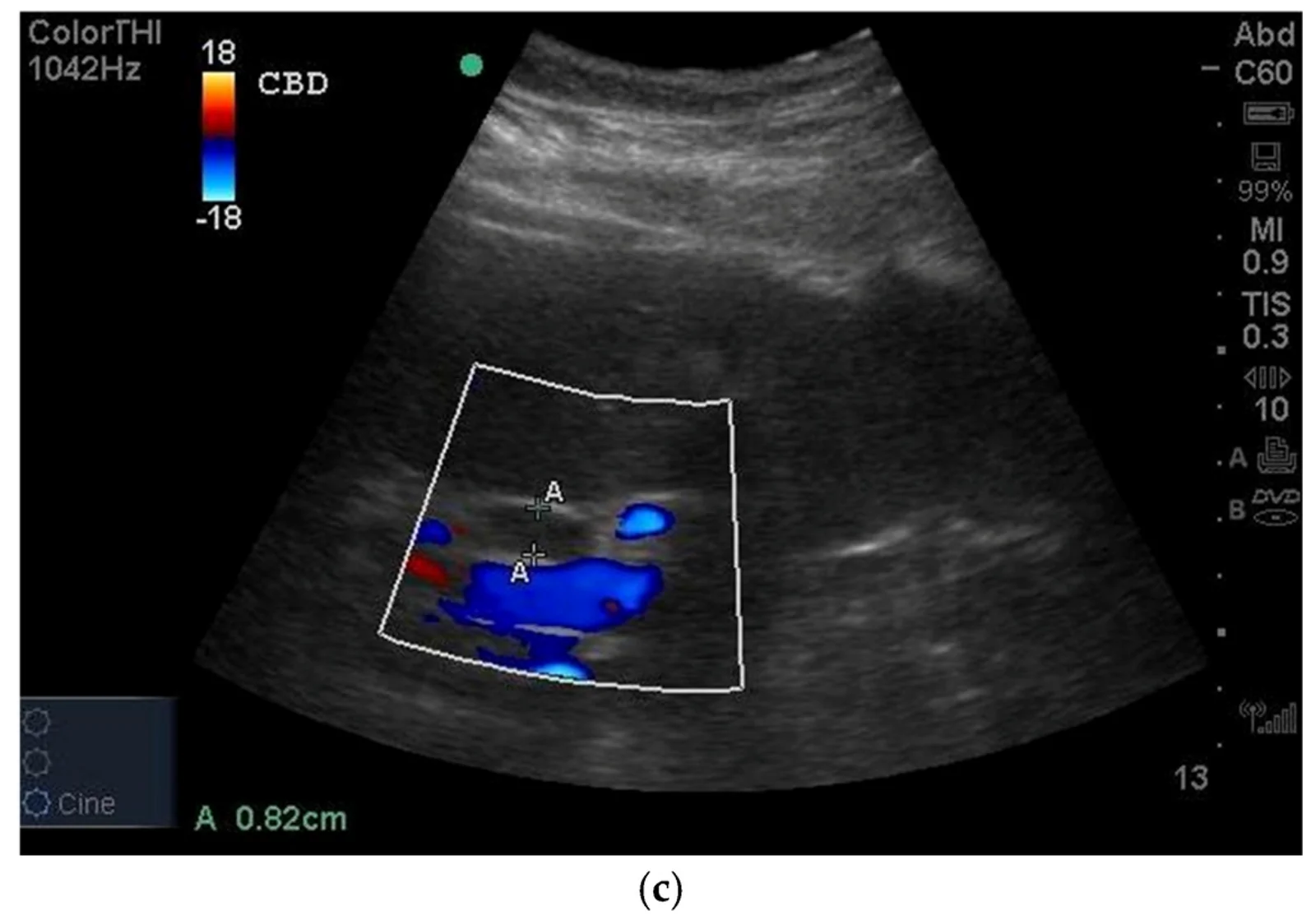
Abdominal pain is one of the most common presenting emergency department (ED) complaints [1,2,3,4]. Hospitalized patients can also develop acute abdominal pain that requires evaluation [4,5,6]. Emergency medicine and critical care society guidelines emphasize ultrasound as a first-line imaging tool for patients with right upper quadrant (RUQ) abdominal pain [7,8,9,10]. Comprehensive RUQ radiology ultrasound can detect biliary pathology involving the gallbladder and common bile duct (CBD). Yet point-of-care ultrasound (POCUS) can be performed at the patient’s bedside for expedited diagnosis and to help guide further imaging, including decisions for subsequent radiology ultrasound or computed tomography (CT). POCUS is operator-dependent and serves as a limited exam to answer specific questions to guide management. POCUS can also facilitate surgical consults if acute cholecystitis or other emergent diagnoses are visualized [7,8,9,10]. Computed tomography may be preferred in patients with diffuse pain, abdominal pain in other locations, or in complicated patients with prior surgeries and altered anatomy. Yet CT scans are sometimes overutilized in EDs and inpatient settings, creating workflow inefficiencies [2,3,4,10,11]. Potential POCUS advantages include avoiding patient exposure to ionizing radiation, mitigating potential safety risks with transport of unstable patients, and lower throughput costs with expedited image acquisition and interpretation times [7,8,9,10]. This Interesting Images publication is a narrative review describing emergent and non-emergent POCUS biliary pathologic findings and presents possible clinical diagnostic pathways for guiding decisions for additional imaging. We searched PubMed, Embase, and Cochrane Reviews for publications written in English from any time period using the search terms “emergent biliary pathology”, “biliary point-of-care ultrasound”, “gallbladder POCUS”, and “common bile duct ultrasound”. We selected publications that were recent (e.g., within 10 years of publication) and high-quality including meta-analyses, systematic reviews, and large prospective studies. From these, we selected high-yield pathologies to present an illustrative image series of cholelithiasis, acute cholecystitis, emphysematous gallbladder, choledocholithiasis or sludge, Mirizzi syndrome, post-operative biliary obstruction, gallbladder polyp, and biliary neoplasia and malignant-appearing lesions. To review gallbladder anatomy, the gallbladder is located underneath the liver and has a body and neck. The neck connects to the cystic duct which drains into the CBD. Two techniques for performing a biliary POCUS exam are described in the literature [1,2,3,4]. Both methods can be used clinically, as one may have better image visualization based on body habitus or gas. (**a**) The X shows the probe placement for both methods. To view the gallbladder with the “X + 7” technique, the user should place the curvilinear probe at the RUQ in transverse position approximately 7 cm to the left of the xiphoid process. The probe marker points to the patient’s right. Tilt the probe, scanning from superior to inferior so that the entire gallbladder is visualized and save the image video clip. Then rotate the probe 90 degrees and repeat to capture the gallbladder in the perpendicular axis. Slowly apply graduated pressure to displace bowel gas for improved viewing if needed. This technique can be used in patients who cannot move their arm to access the mid-axillary line view, who are lying supine, and who are able to turn to the left lateral decubitus position (LLD) for improved structure visualization. (a) The asterisk shows an alternate method of performing biliary POCUS in patients with severe RUQ pain or nausea who cannot tolerate the probe pressure. It can also be used in patients with limited anterior views due to bowel gas, obesity or a distended abdomen, and in patients who can rotate to the LLD position. Place the curvilinear ultrasound probe at the right mid-axillary line at the inferior aspect of the ribs and slide the probe anteriorly until the gallbladder is visualized. Tilt the probe from anterior to posterior so that the entire gallbladder is visualized. Rotate the probe 90 degrees and repeat, tilting from superior to inferior to visualize the gallbladder in the perpendicular axis. To measure the gallbladder wall, reduce the image depth to visualize the anterior wall in either view and save the measurement. Normal gallbladder wall thickness is 3 mm or less [1,2,3,4]. (**b**,**c**) To view the CBD, scan through the gallbladder in longitudinal view (body appears elongated) and past the neck of the gallbladder. Posterior to the gallbladder will be a larger vessel (portal vein) and two smaller adjacent veins (hepatic artery and common bile duct). This is sometimes called the “mickey mouse sign” as an orientation aid. For the perpendicular view, rotate the probe 90 degrees and scan posteriorly until the CBD is visualized running longitudinally on top of the portal vein. The CBD should not have flow with color Doppler mode, which helps distinguish it from the hepatic artery [1,2,3,4].

**Figure 2:**
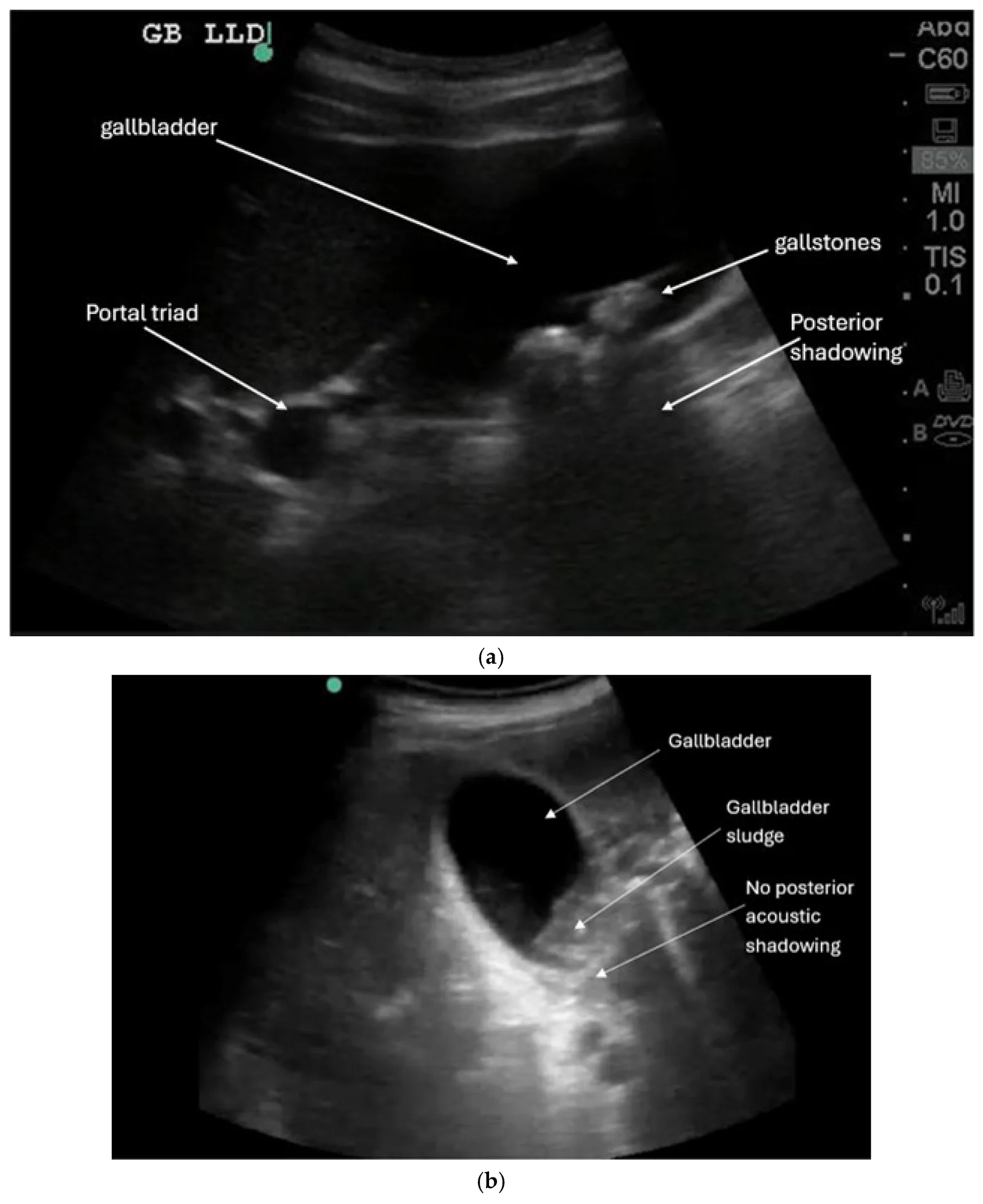
(**a**,**b**) Cholelithiasis is defined by the presence of gallstones within the gallbladder. It usually presents asymptomatically or with biliary colic pain when transient obstruction of the cystic duct occurs, often triggered by fatty meals [1,2,5,6]. POCUS has high sensitivity (96–97%) and specificity (97–99%) for detecting gallstones that are >1.5 mm diameter in size [5,6]. Using a curvilinear probe in the RUQ, the clinician should tilt the probe to view the entire gallbladder in a transverse plane. Then rotate the probe 90 degrees to view the gallbladder in a longitudinal axis. On ultrasound, gallstones usually appear as small or large round hyperechoic mobile structures with posterior shadowing due to their increased density (**a**). A video clip should be obtained in the LLD position to ensure gallstone mobility with patient position changes [3,4,5]. In (**a**), the portal triad is also visible, which contains the hepatic artery, portal vein, and the common bile duct. (**b**) is an example of gallbladder sludge, which can be present with or without gallstones. Sludge can be distinguished from stones because it does not have posterior acoustic shadowing or mobility. Patients with symptomatic cholelithiasis with severe pain and vomiting may warrant a surgical consult for admission. Otherwise, they can be evaluated outpatient with a general surgery referral for possible outpatient cholecystectomy.

**Figure 3:**
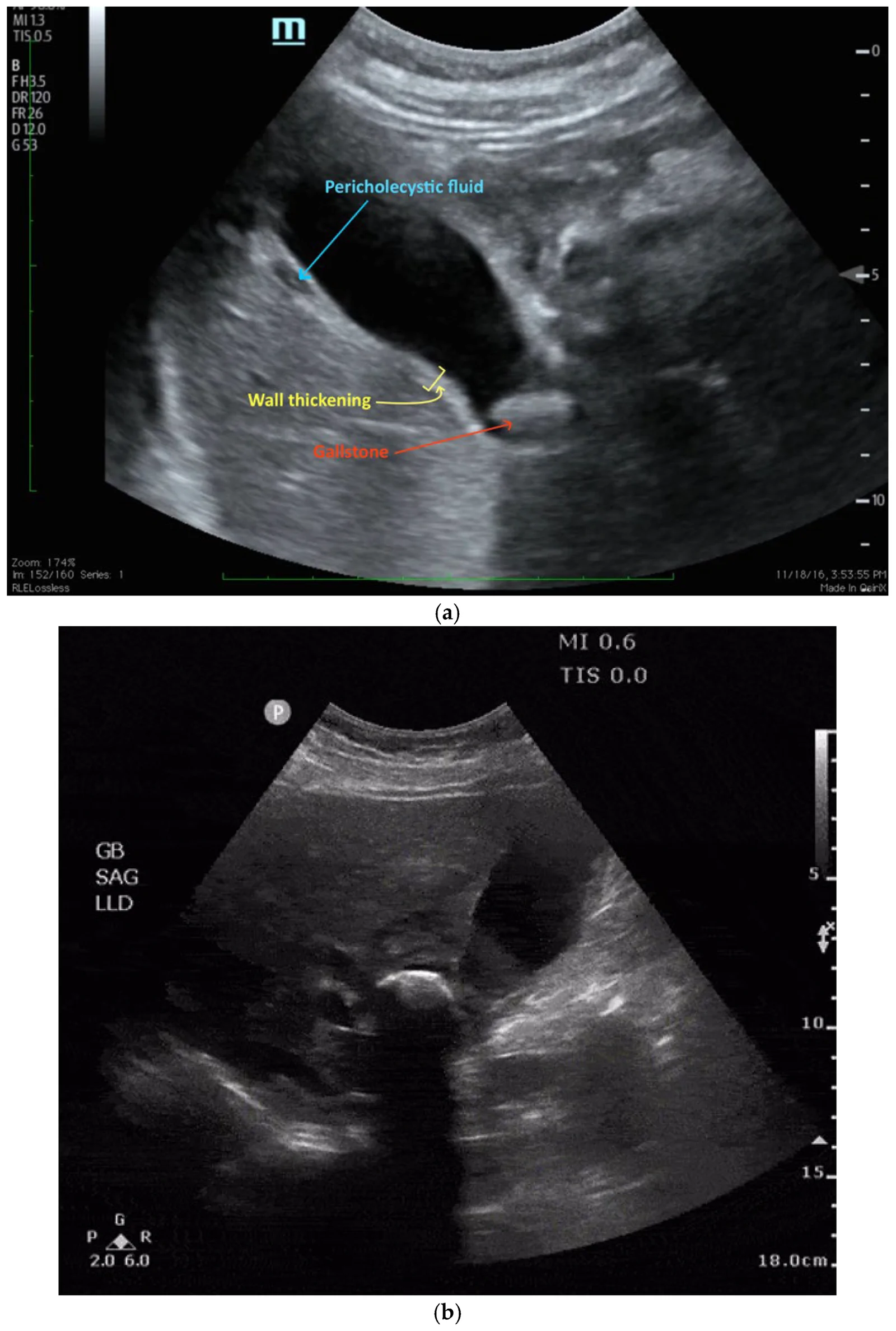
(**a**,**b**) Obstruction of the cystic duct by a gallstone can cause gallbladder inflammation and can develop into acute cholecystitis. Cholelithiasis causing obstruction at the gallbladder neck is the most common cause of acute cholecystitis, which accounts for up to 9% of hospital admissions for abdominal pain [7]. For detection of acute cholecystitis, POCUS accuracy varies across studies and has ranges reported as 71–88% sensitivity and 80–100% specificity [3,4,8,9,10,11]. Moreover, a systematic review and meta-analysis performed in 2024 across 7 databases compared emergency physicians performing POCUS against a reference standard of final diagnosis (surgical pathology, discharge diagnosis, and radiology-performed ultrasound). They found a pooled sensitivity of 70.9% (95% confidence interval [CI] 62.3 to 78.2), specificity of 94.4% (95% CI 88.2 to 97.5), positive likelihood ratio of 12.7 (5.8 to 27.5), and negative likelihood ratio of 0.31 (0.23 to 0.41) for the diagnosis of acute cholecystitis [7]. Based on this performance data, POCUS is useful for detecting disease, but a negative or technically limited scan does not exclude acute cholecystitis when clinical suspicion remains high. Key identifying factors on ultrasound include gallbladder wall thickening greater than 3 mm (measured anteriorly), presence of pericholecystic fluid, and a positive sonographic Murphy’s sign. This is defined as pain elicited with probe pressure over the gallbladder and lack of pain with the probe at another site [3,4,9,10]. (**a**) is an example of an obstructing calculus causing gallbladder inflammation with pericholecystic fluid and gallbladder wall thickening. (**b**) is an example of a stone in the gallbladder neck that is causing external compression of the biliary tree, which is a less common disease known as Mirizzi syndrome. Prolonged stone immobility can cause chronic inflammation and fibrosis [3,4]. In cases that are equivocal for acute cholecystitis (only meets 1–2 of the above diagnostic imaging criteria) or symptomatic cholelithiasis, surgical consultants may request additional imaging. This could be a RUQ radiology ultrasound, CT scan, or a hepatobiliary iminodiacetic acid (HIDA) scan to evaluate for complete gallstone obstruction of the cystic duct for emergent surgical planning. A HIDA scan is appropriate for patients with high clinical suspicion of cholecystitis but equivocal ultrasound findings [12]. With acute cholecystitis, the HIDA scan will show no nuclear tracer uptake into the gallbladder within 1–4 h due to an obstructed cystic duct. Whereas, CT scan is better for complicated or post-operative patients with altered anatomy [12]. In a study by Oda et al. comparing CT scan and ultrasound for diagnosis of acute cholecystitis, CT was recommended as a follow up study for equivocal cases with high sensitivity 85% and specificity 100% [10].

**Figure 4:**
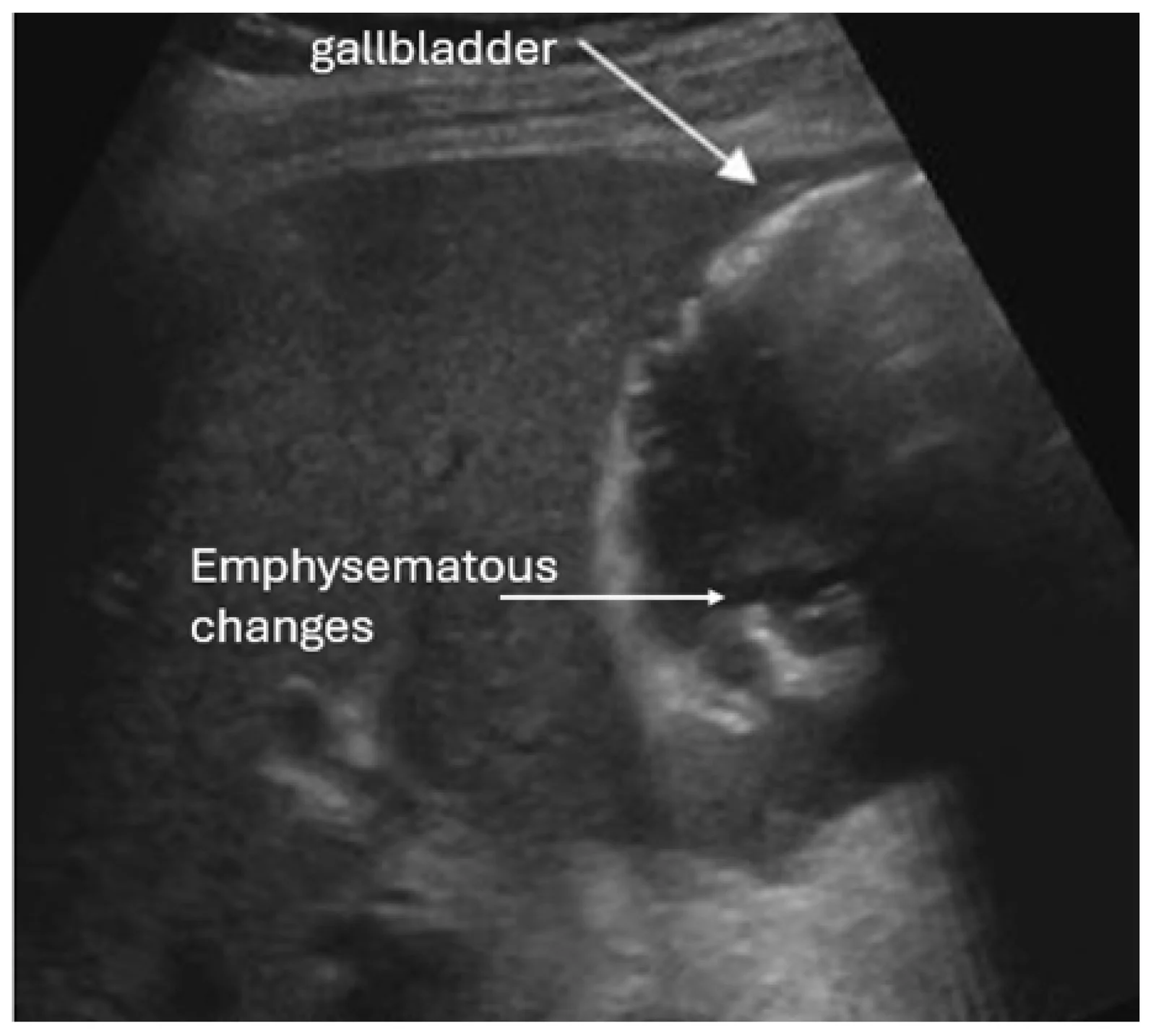
Another serious gallbladder complication is emphysematous cholecystitis, caused by bacterial superinfection of the gallbladder wall with gas-producing organisms. Diabetes mellitus with hyperglycemia is a significant risk factor, and the gallbladder can become ischemic with poor vascular flow [3,4]. Ultrasound characteristics include presence of air within the gallbladder wall, which appears as hyperechoic foci with associated reverberation or “dirty shadowing” artifact [3,4,13,14,15]. Ultrasound is highly specific for detecting air within the gallbladder wall, though sensitivity is decreased due to air interfering with clear visualization of the gallbladder [13,14,15]. Figure 4 is an example of emphysematous cholecystitis with free air within the gallbladder wall and posterior “dirty shadowing”. A CT scan should be performed if ultrasound images are not clear. When emphysematous cholecystitis is diagnosed, physicians should emergently consult general surgery and initiate intravenous antibiotics.

**Figure 5:**
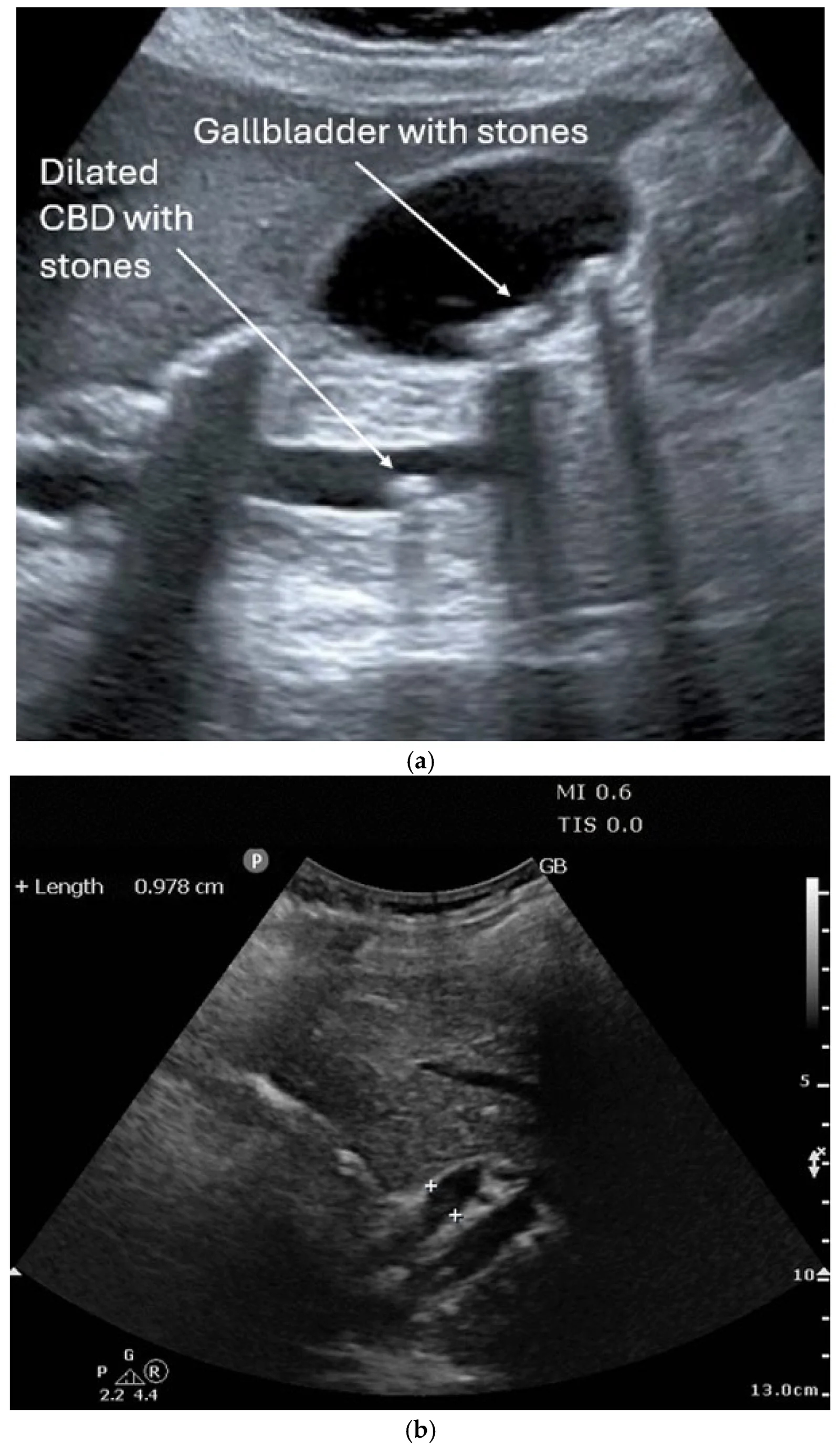
(**a**,**b**) Gallstones can escape the gallbladder through the ductal system and cause choledocholithiasis, which refers to stones within the CBD. Patients often present with colicky RUQ abdominal pain, with possible jaundice, pruritis, nausea, and vomiting. When assessing for choledocholithiasis, POCUS should be performed both longitudinally and transversely through the common bile duct. Radiology defines a dilated CBD as >6 mm. For POCUS users, a practical reference for calculating a normal CBD diameter is 3 mm plus 1 mm for every decade in age over 30 years old, although this is not an absolute diagnostic threshold [3,4]. Exceptions to this rule include post-cholecystectomy patients, whose CBD may measure up to 10 mm and patients with older age, who may have a higher CBD measurement. Potential CBD findings include visualization of intraluminal stones as hyperechoic round structures ranging from 2 mm to over 20 mm [3,4,16,17]. Users should note that gas in the duodenum may complicate stone detection, so they should be cautious with a negative POCUS exam but high clinical suspicion. The common bile duct and intrahepatic biliary tree may be dilated without a visualized stone. Additionally, multiple stones in the gallbladder may be visualized, which should increase suspicion for choledocholithiasis. In these cases, particularly if the patient has transaminitis or an elevated lipase level, additional workup should be considered such as a RUQ radiology ultrasound. A CT scan can be considered for a comprehensive view of the gallbladder and for ductal dilation, plus complications such as pancreatitis or adjacent abscess. Gastroenterology should be consulted for potential intervention with Endoscopic Retrograde Cholangiopancreatography (ERCP) to remove the stones or for Magnetic Resonance Cholangiopancreatography (MRCP) to visualize and confirm their location. Complications from choledocholithiasis can include ascending cholangitis, appearing as bile duct wall thickening on POCUS. In cases of ascending cholangitis, the patient can become critically ill quickly. Thus, they should be treated emergently with intravenous antibiotics to prevent serious complications and mortality from infection dissemination [3,4,16,17]. Furthermore, patients with prior cholecystectomy or RUQ abdominal surgery can develop post-operative biliary obstruction. This can sometimes be visualized on ultrasound as biliary ductal dilation with possible extension into the hepatic ducts. Post-operative cholecystectomy patients can have normal CBD dilation up to 10 mm. Recent surgery warrants general surgery consultation and CT imaging for further clarity, as POCUS performance in the post-operative patient is very limited [3,4,16,17]. Due to post-surgical changes in anatomy, cross-sectional imaging should be prioritized in these patients. CT can provide a rapid assessment of post-operative anatomy and complications. Whereas, magnetic resonance imaging (MRI)/MRCP offers a more detailed view of the biliary and pancreatic ductal systems [17,18].

**Figure 6:**
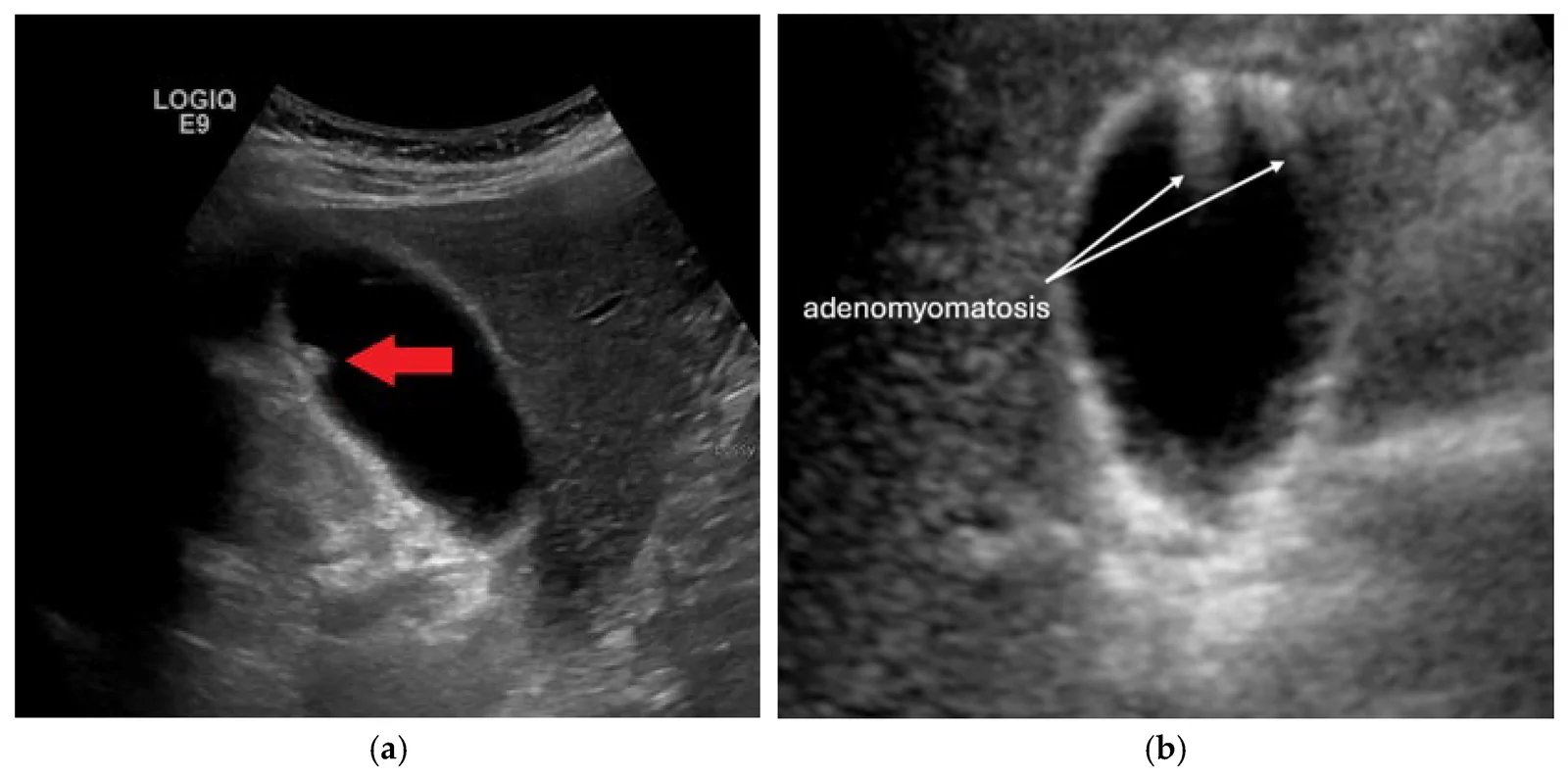
(**a**,**b**) While performing POCUS, users should be aware of other non-emergent biliary findings that may be seen. Gallbladder polyps are often asymptomatic and are common incidental findings on abdominal ultrasound. They are divided into pseudopolyps (more common and benign) or true polyps [19,20]. Pseudopolyps include focal adenomyomatoses and cholesterol polyps. True polyps can be benign or malignant and are defined as adenomas or adenocarcinomas [19]. True polyps, as seen in (**a**), will appear as a small, hyperechoic, non-motile outgrowth of the gallbladder wall that can be pedunculated or sessile, (red arrow) whereas pseudopolyps will display a “comet-tail” artifact [21]. Clinicians should perform a dynamic POCUS assessment to differentiate between biliary sludge and polyps, as sludge is motile and will mobilize off the gallbladder wall when the patient rotates from supine to the LLD position [8,20]. Adenomyomatosis is a type of pseudopolyp that is often benign and characterized by excessive growth of the gallbladder wall that can progress into mural diverticula known as Rokitansky–Aschoff sinuses (RAS). These formations appear as intramural anechoic spaces on POCUS and when laden with calculi or cholesterol crystals generate a “comet-tail” artifact (**b**). This is due to the ultrasound beam generating reverberation artifact off the aggregates within the RAS [8,20,22,23]. The comet-tail artifact is typical but not unique to adenomyomatosis, as it is also seen in cholesterol polyps [19,22,23]. Patients with benign findings typically do not require acute intervention and can follow up for surveillance or further outpatient imaging with a general surgery referral.

**Figure 7:**
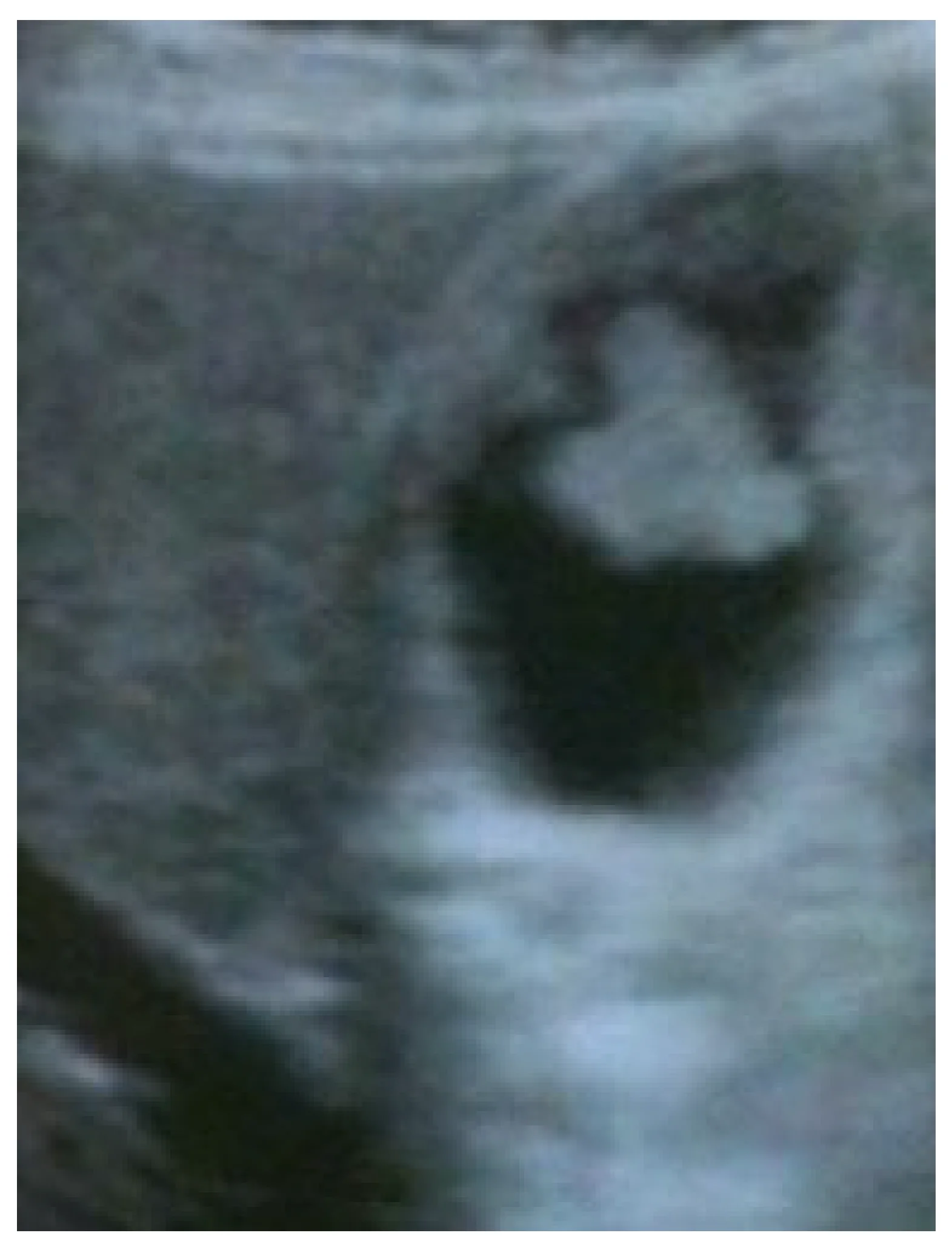
Gallbladder neoplasia is an uncommon cancer that can form from a benign polyp with high-risk features progressing into a malignant mass (e.g., large size > 10 mm with adjacent wall thickening). Gallbladder tumors are often detected incidentally during cholecystectomy or on gallbladder imaging [24,25,26,27]. Surgical pathology is needed for a definitive diagnosis, but ultrasound can be used to visualize and characterize the mass. Figure 7 shows a longitudinal view of a malignant mass present in the gallbladder. Ultrasound is the first-line imaging test for gallbladder malignancies and is used for (1) risk stratification of wall thickening and (2) polypoidal types of gallbladder malignancies. Gallbladder polyps are classified into three categories based on morphologic features and size. Extremely low-risk polyps are pedunculated with a “ball-on-the-wall” character or a thin stalk. Low risk polyps are pedunculated with a thick or wide stalk or a sessile character. Intermediate-risk polyps have adjacent focal wall thickening. A larger sized gallbladder polyp is the strongest predictor for malignancy (e.g., over 10 mm) [24,25,26,27]. In a systematic review, the positive predictive value of transabdominal ultrasound for detecting neoplastic polyps was 4.5% and 1.4% [23]. Yet POCUS users should be cautious because other reviews by Li et al. and Metman et al. found a high false positive rate of 72.5% and 95.4% for transabdominal ultrasound detecting neoplastic polyps [23]. Thus surgical decisions should be based on individual cases and may need additional CT or MRI [21]. Detailed evaluation of gallbladder wall features also helps risk stratify gallbladder malignancies [25,26,27]. A recent systematic review found that presence of echogenic foci, lack of wall disruption, and hypoechoic nodules are associated with benign thickening, while focal thickening and indistinct interface were associated with malignant wall thickening [24,25,26,27]. If the symptoms are debilitating (e.g., severe pain, intractable vomiting, cannot tolerate oral intake, etc.) then the patient may need surgical consultation and hospital admission. Otherwise, they should be referred to oncology for outpatient testing and management.

**Figure 8:**
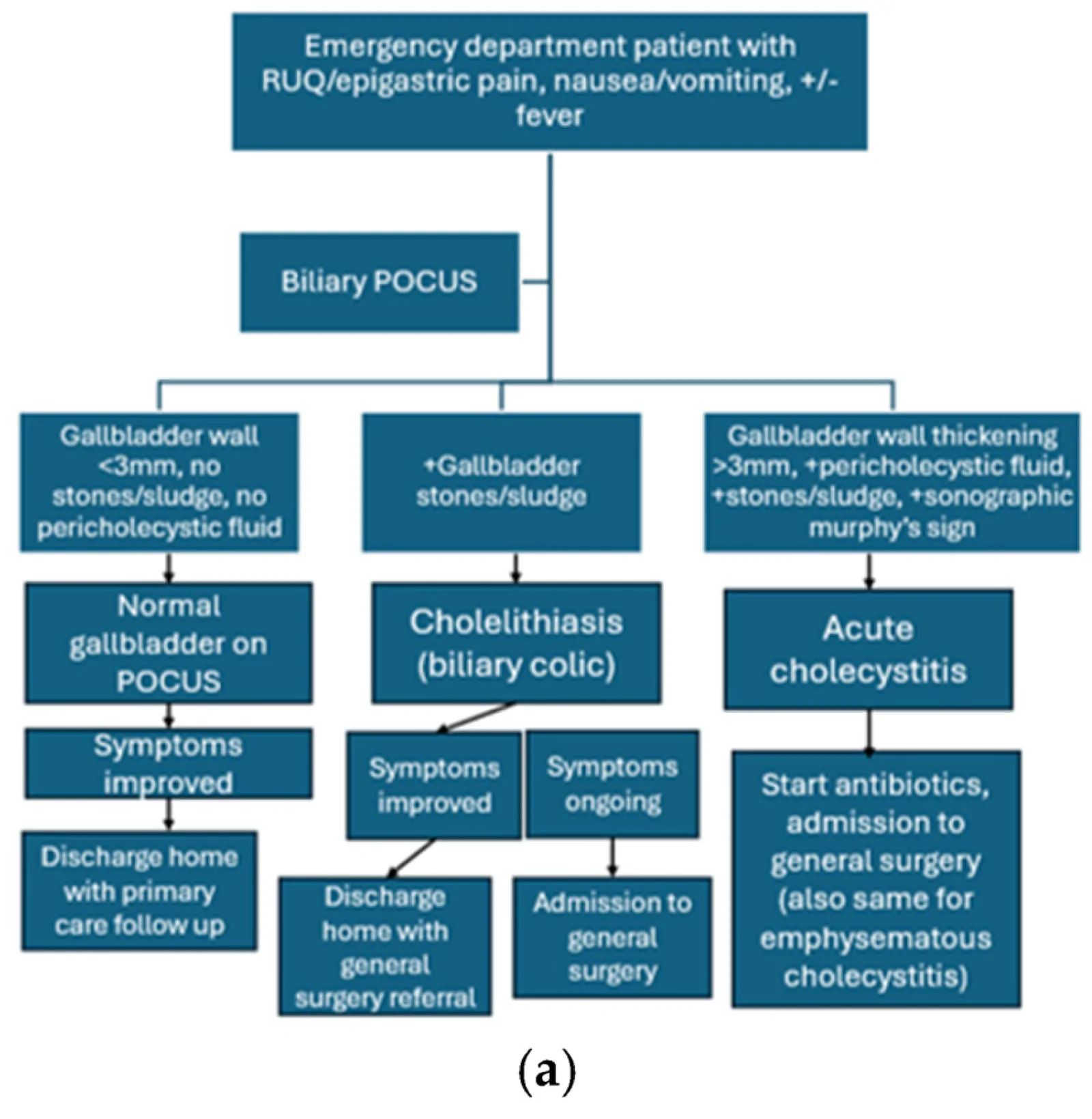

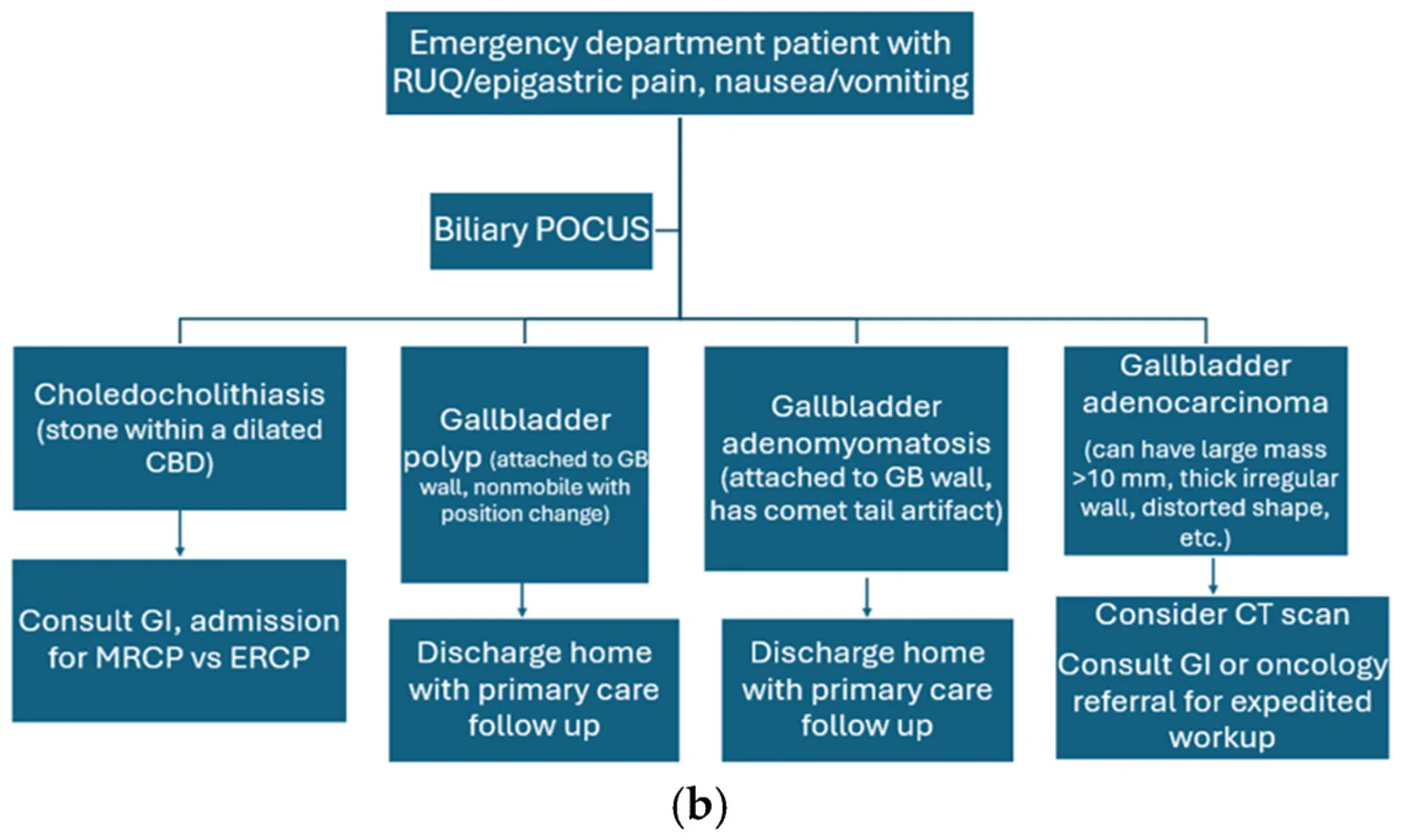

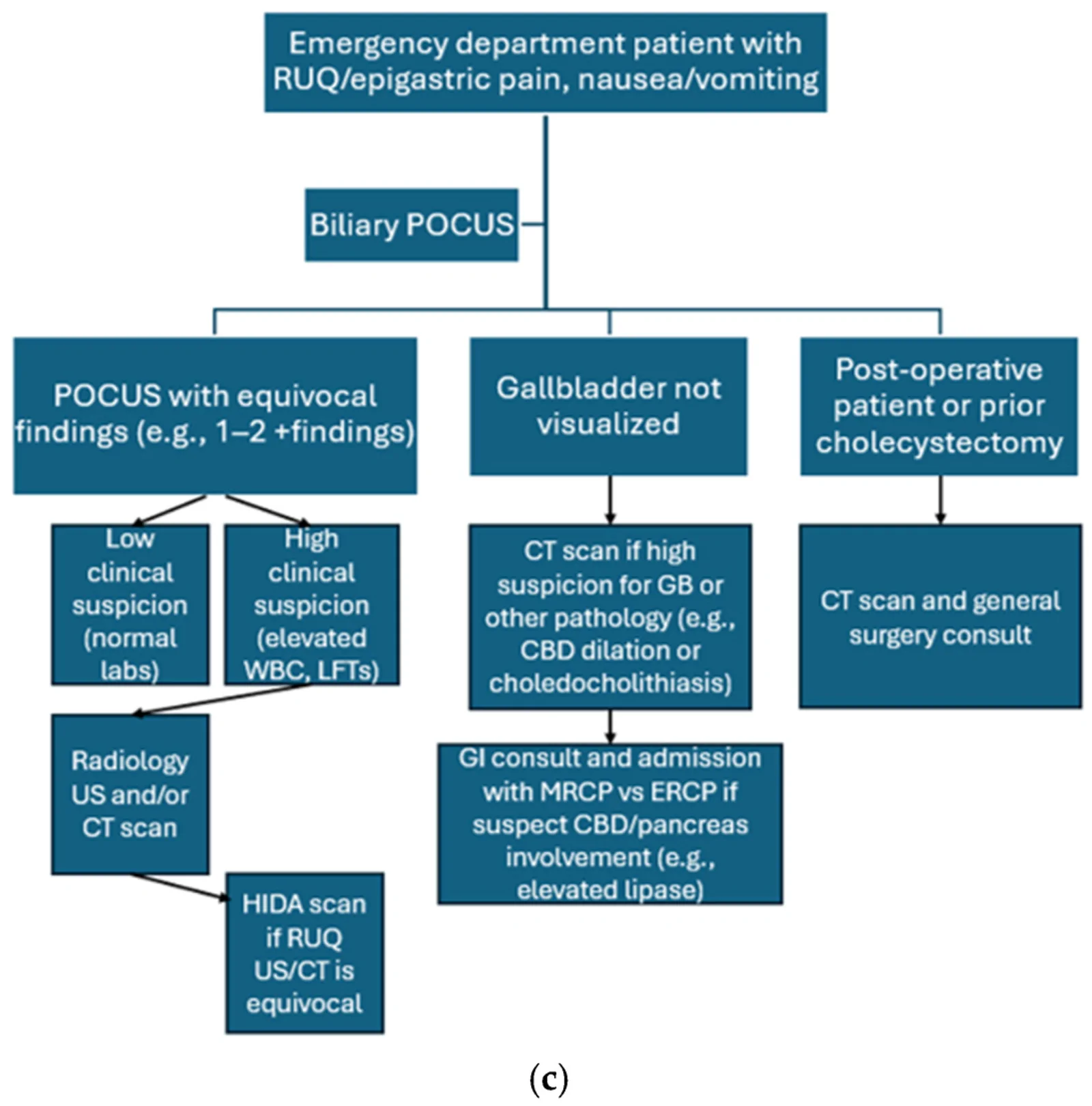
(**a**–**c**) To summarize the clinical biliary diagnostic pathway, we propose suggested clinical pathways for emergent and non-emergent biliary diagnosis and treatment (Supplemental Table S1). Users should utilize POCUS as the first-line imaging modality for ED and hospitalized patients presenting with acute RUQ abdominal pain. POCUS integration into existing clinical diagnostic pathways can expedite diagnosis of biliary disease and guide further imaging and treatment decisions. Although POCUS is a rapid diagnostic bedside exam, sometimes the results are equivocal, which requires subsequent RUQ radiology ultrasound, CT scan, or HIDA scan. Subsequent imaging and specialist referral should also take into account pre-test probability, local expertise, institutional protocols, and resource availability. In cases where the gallbladder cannot be visualized or the patient is post-operative, subsequent CT imaging is usually necessary if there is high clinical suspicion for biliary or other pathology. General surgery should be consulted for patients with acute cholecystitis, symptomatic cholelithiasis, or emphysematous cholecystitis. Surgical consultants may request a HIDA scan in cases of equivocal cholecystitis to confirm there is no nuclear tracer uptake into the gallbladder within 1–4 h due to an obstructed cystic duct for emergent surgical planning. Whereas, gastroenterology will treat patients endoscopically for choledocholithiasis. Finally, patients who are critically ill with complications such as gallbladder perforation, abscess, ascending cholangitis, etc., and who cannot safely undergo surgery should be admitted to the hospital for intravenous antibiotics and additional resuscitative care. Drain placement by interventional radiology is sometimes an option in these patients to help decompress the inflammation and for fluid collections. Supplemental Table S2 summarizes the major diagnostic ultrasound findings for each biliary pathology described. Supplemental Table S3 describes the sensitivity and specificity of ultrasound compared to CT for various biliary pathologies. Values are provided as a range due to study heterogeneity, and the results are limited by the existing literature. In conclusion, this narrative review illustrates important diagnostic findings for major emergent and non-emergent biliary diseases and emphasizes the clinical application of POCUS to guide decisions on radiology ultrasound, CT imaging, and MRCP (**a**–**c**). In turn the suggested pathways can guide treatment including expedited surgical and gastroenterology consults.

## Data Availability

The original contributions presented in this study are included in the article. Further inquiries can be directed to the corresponding author.

## Supplementary Materials

The following supporting information can be downloaded at: https://www.mdpi.com/article/10.3390/diagnostics16183023/s1, Video S1: longitudinal view of a normal gallbladder. Video S2: longitudinal view of the common bile duct; Video S3: hyperechoic gallstones with posterior shadowing. Video S4: large gallbladder stone in the neck with posterior shadowing. Video S5: acute cholecystitis with wall thickening, pericholecystic fluid, and gallstones. Video S6: emphysematous cholecystitis with air in the gallbladder wall and dirty shadowing. Table S1: suggested clinical biliary diagnostic pathway based on point-of-care ultrasound findings. Table S2: major diagnostic findings for each biliary pathology described. Table S3: sensitivity and specificity of ultrasound compared to computed tomography (CT) for various biliary pathologies.

## Abbreviations

The following abbreviations are used in this narrative review:

POCUS	Point-of-care ultrasound
ED	Emergency department
RUQ	Right upper quadrant
RAS	Rokitansky–Aschoff sinuses
ERCP	Endoscopic Retrograde Cholangiopancreatography
MRCP	Magnetic Resonance Cholangiopancreatography
MRI	Magnetic resonance imaging
WBC	White blood cell count
LFTs	Liver function tests
HIDA	hepatobiliary iminodiacetic acid
