# Supplementary material for: Acute Emergent Biliary Pathologies Identified Using Point-of-Care Ultrasound
Source: Diagnostics (Basel). 2026 Sep 17;16(18):3023. doi: 10.3390/diagnostics16183023 (PMC13605488; doi:10.3390/diagnostics16183023)
Supplement: Supplementary file 1 [file diagnostics-16-03023-s001.zip › Supplemental Table S3 Biliary.pdf]

Supplemental Table S3. Sensitivity and specificity of ultrasound compared to computed tomography (CT) for various biliary pathologies [8,10–13,15,16,20–22].

Biliary pathology	Ultrasound sensitivity	Ultrasound specificity	Contrast-enhanced CT sensitivity	Contrast-enhanced CT specificity
Cholelithiasis	96, 97% (95% CI 93.6% to 99.5%)	99.5% (95% CI 97.3% to 99.99%)	94%	59%
Acute cholecystitis	70.9, 72, 79, 81, 83.8, 88% (95% 62.3 to 91.6%)	80, 83, 94.4, 98.6, 100% (95% CI 88.2 to 99.6%)	85, 92, 94, 100%	85-100%
Choledocholithiasis	79%	53%	50%–80%	57%–71%
Gallbladder polyp (>10 mm)	100%	86%	9%	
Gallbladder malignancy	76% standard ultrasound; 97% (endoscopic ultrasound)	36-100%	73%	90%
