# Supplementary material for: Acute Emergent Biliary Pathologies Identified Using Point-of-Care Ultrasound
Source: Diagnostics (Basel). 2026 Sep 17;16(18):3023. doi: 10.3390/diagnostics16183023 (PMC13605488; doi:10.3390/diagnostics16183023)
Supplement: Supplementary file 1 [file diagnostics-16-03023-s001.zip › Supplemental Table S1 Biliary.pdf]

Supplemental Table S1. Suggested clinical biliary diagnostic pathway based on point-of-care ultrasound findings

POCUS findings	Biochemical findings	Clinical features	Low/intermediate/ high probability	Next test
Normal gallbladder	Normal liver enzymes and lipase, normal WBC	Abdominal pain, nausea/vomiting	Low →<br>Intermediate →<br>High →	-None<br>-RUQ US<br>-RUQ US or CT scan
Equivocal GB = (gallstones or sludge, wall thickening)	Normal liver enzymes and lipase, normal WBC	RUQ pain, nausea/vomiting	Low →<br>Intermediate →<br>High →	-HIDA scan or RUQ US<br>-RUQ US or CT scan
Gallstones or sludge, GB wall thickening >3 mm, pericholecystic fluid, +sonographic murphy's sign	Transaminitis, leukocytosis	RUQ pain, nausea/vomiting	Low →<br>Intermediate →<br>High →	Consult general surgery; RUQ US or CT scan if requested
Dilated CBD, gallstone in the CBD	Transaminitis, elevated lipase	RUQ/epigastric pain, nausea/vomiting	Low →<br>Intermediate →<br>High →	-RUQ US<br>-RUQ US<br>-consult GI, ERCP/MRCP
Emphysematous GB, gas foci	Transaminitis, leukocytosis, elevated lactate	RUQ pain, nausea/vomiting, fever	Low →<br>Intermediate →<br>High →	-Consult general surgery; CT scan if requested
No gallbladder visualized	Normal or elevated liver enzymes/lipase	RUQ pain, nausea/vomiting	Low →<br>Intermediate →<br>High →	-CT scan for post-operative patients
GB polyp or adenomyomatosis	Normal liver enzymes and lipase, normal WBC	RUQ pain	Low →<br>Intermediate →<br>High →	-None, outpatient follow up<br>-RUQ US
GB large mass, wall thickening	Normal liver enzymes and lipase, normal WBC	RUQ pain, nausea/vomiting	Low →<br>Intermediate →<br>High →	None, outpatient f/u<br>-RUQ US or CT scan
Normal gallbladder	Normal liver enzymes and lipase, normal WBC	Abdominal pain, nausea/vomiting	Low →<br>Intermediate →<br>High →	-None<br>-RUQ US<br>-RUQ US or CT scan

Key: GB=gallbladder, CBD=common bile duct, WBC=white blood cell count, RUQ=right upper quadrant, US=ultrasound, CT=computed tomography, HIDA= hepatobiliary iminodiacetic acid, ERCP=Endoscopic Retrograde Cholangiopancreatography, MRCP=Magnetic Resonance Cholangiopancreatography, f/u=follow up, GI=gastroenterology
