# Supplementary material for: Acute Emergent Biliary Pathologies Identified Using Point-of-Care Ultrasound
Source: Diagnostics (Basel). 2026 Sep 17;16(18):3023. doi: 10.3390/diagnostics16183023 (PMC13605488; doi:10.3390/diagnostics16183023)
Supplement: Supplementary file 1 [file diagnostics-16-03023-s001.zip › Supplemental Table S2 Biliary.pdf]

Supplemental Table S2. Major diagnostic findings for each biliary pathology described

Biliary pathology	Diagnostic findings on ultrasound
Cholelithiasis	Hyperechoic round stones within the gallbladder
Acute cholecystitis	Wall thickening >3mm, pericholecystic fluid, gallstones
Emphysematous cholecystitis	Anechoic gas with dirty shadowing, thickening gallbladder wall >3mm, pericholecystic fluid
Choledocholithias is	Hyperechoic round stones within the common bile duct
Gallbladder polyp	Hyperechoic structure attached to the gallbladder wall, not mobile with position changes
Gallbladder malignancy	Abnormal echotexture of gallbladder and wall, visualized mass, distortion of normal structures
